# Open source machine-learning algorithms for the prediction of optimal cancer drug therapies

**DOI:** 10.1371/journal.pone.0186906

**Published:** 2017-10-26

**Authors:** Cai Huang, Roman Mezencev, John F. McDonald, Fredrik Vannberg

**Affiliations:** 1 School of Biological Sciences, Georgia Institute of Technology, Atlanta, Georgia, United States of America; 2 Parker H. Petit Institute for Bioengineering and Bioscience, Georgia Institute of Technology, Atlanta, Georgia, United States of America; Harbin Institute of Technology Shenzhen Graduate School, CHINA

## Abstract

Precision medicine is a rapidly growing area of modern medical science and open source machine-learning codes promise to be a critical component for the successful development of standardized and automated analysis of patient data. One important goal of precision cancer medicine is the accurate prediction of optimal drug therapies from the genomic profiles of individual patient tumors. We introduce here an open source software platform that employs a highly versatile support vector machine (SVM) algorithm combined with a standard recursive feature elimination (RFE) approach to predict personalized drug responses from gene expression profiles. Drug specific models were built using gene expression and drug response data from the National Cancer Institute panel of 60 human cancer cell lines (NCI-60). The models are highly accurate in predicting the drug responsiveness of a variety of cancer cell lines including those comprising the recent NCI-DREAM Challenge. We demonstrate that predictive accuracy is optimized when the learning dataset utilizes all probe-set expression values from a diversity of cancer cell types without pre-filtering for genes generally considered to be “drivers” of cancer onset/progression. Application of our models to publically available ovarian cancer (OC) patient gene expression datasets generated predictions consistent with observed responses previously reported in the literature. By making our algorithm “open source”, we hope to facilitate its testing in a variety of cancer types and contexts leading to community-driven improvements and refinements in subsequent applications.

## Introduction

The sequencing of the human genome, genome-wide association studies (GWAS), quantitative trait loci (QTL) mapping, and similar research initiatives over the past few decades have greatly increased our understanding of the molecular pathways associated with human diseases. These efforts have significantly benefited from the liberal sharing of data and open-source scripts utilized for these efforts. Over the last few years, there has been a number of alternative machine-learning (ML) approaches employed in personalized cancer drug prediction, each associated with variable degrees of success [1–3]. For example, pRRocphetic [4] is a recently designed R package designed to run the entire learning and subsequent calling of patient data. Other recent contributions include the Bioconductor [5] package SCAN that allows for single-sample array normalization for precision medicine workflows. While a number of ML applications for precision medicine have benefited from community assessments of predicted drug response [*e*.*g*., [1,2]), such efforts have not always shared code, and for the majority of efforts only the organizers of the community assessment exercise were able to see the source code to evaluate each independent solution. This is unfortunate because the open sharing of code has been demonstrated to be a significant catalyst in the optimization of ML applications as in the Large Scale Visual Recognition Challenge (ILSVRC) where computational solutions are openly available [6,7].

We present here an open source software platform using a highly versatile support vector machine (SVM) algorithm that utilizes standard recursive feature elimination (RFE) methods to predict cancer drug response. In pilot applications, we utilized publicly available datasets from NCBI Gene Expression Omnibus (GEO) [8] that we formatted and the array files were partitioned into learning sets and experimental sets. Each individual array is accessible as a.CEL file with individual identifiers at a publically accessible GitHub site that outlines the learning, validation and test sets employed in our initial studies (https://github.com/chuang95/KEA_DrugResponse). Also available at this GitHub site are general procedures for open application of our software to additional datasets (https://github.com/chuang95/KEA_DrugResponseLearning). We have employed the algorithms to explore the effect of a variety of alternative learning datasets on predictive accuracy leading to several unanticipated findings. First, predictive accuracy was significantly improved when microarray probe level expression data rather than average gene expression values were employed in the model building process. Second, predictive accuracy was improved when models were built upon a diversity of cancer types. Third, the pre-filtering of learning datasets based upon preconceived biological models significantly reduces predictive accuracy. Application of our optimized models to publically available ovarian cancer (OC) patient gene expression datasets generated predictions highly consistent with observed responses to a variety of drugs. By providing true open access to our software, we seek to encourage additional improvements in current methods, as well as, constructive comparisons with alternative approaches leading to the development of optimal ML-based strategies for personalized cancer medicine.

## Results

### Support vector machine (SVM) model building and recursive feature selection

**Fig 1:**
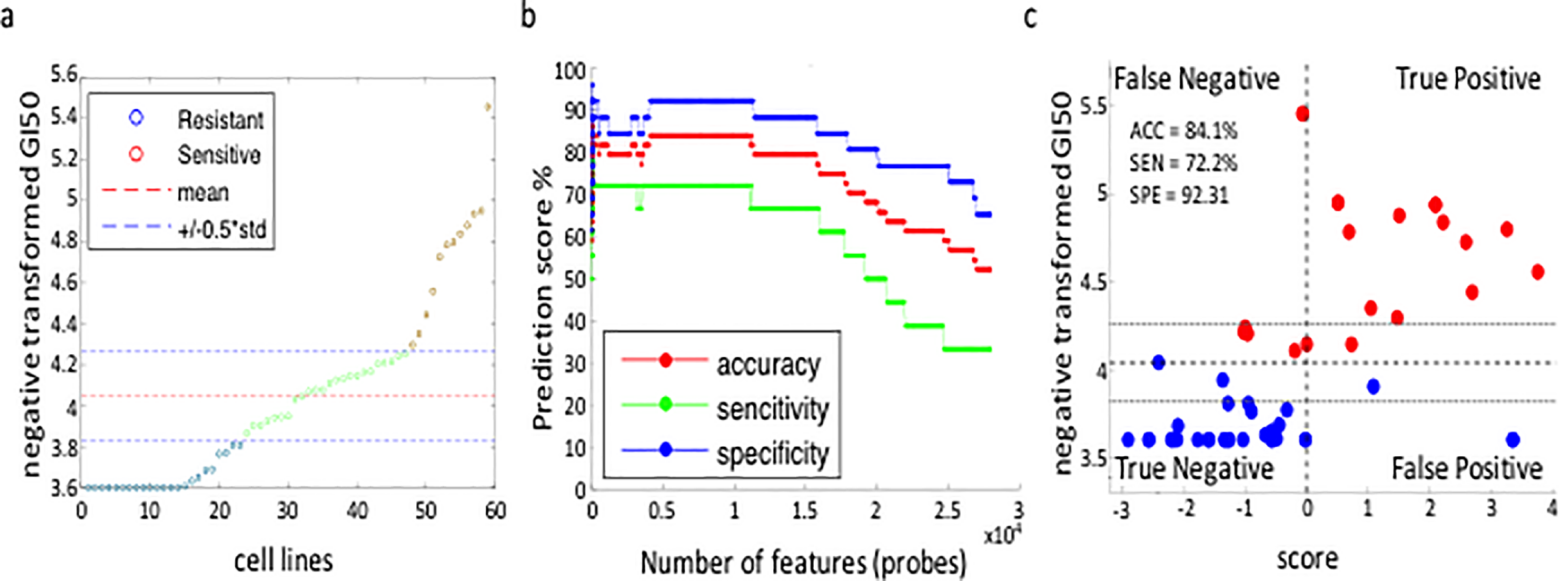
An SVM-RFE predictive model of carboplatin sensitivity for NCI-60 cell lines. (A) Ranked display of -log transformed GI50 values for carboplatin for each of the NCI-60 cell lines. Blue circles = carboplatin resistant cells; red circles = carboplatin sensitive cell lines. Cell lines with GI50 values within ±0.5 SD of the mean (green circles) are less reliably classified as resistant or sensitive and were, thus, not employed in learning datasets. Test sets were selected from cell lines across the entire distribution; (B) Evolution of accuracy of predicted response to carboplatin using SVM-RFE selection for gene probe classifiers; (C) Visualization of the optimal separation between carboplatin sensitive and resistant NCI-60 cell lines. The X-axis is the optimal weight vector (prediction score) of the SVM model for carboplatin; the Y-axis is the -log transformed GI50 values for carboplatin.

A variety of ML techniques and strategies have been employed in the quest for optimal accuracy, sensitivity and specificity in drug response predictions. In this work, we utilize an SVM approach paired with recursive feature elimination (RFE). SVM has been successfully applied in a variety of biological applications in recent years (e.g., [9]). Our SVM models were built using gene expression (https://www.ncbi.nlm.nih.gov/geo/query/acc.cgi?acc=GSE32474) (see also Table A in S2 File) and drug sensitivity profiles (https://wiki.nci.nih.gov/display/NCIDTPdata/NCI-60+Growth+Inhibition+Data)) (see also Table B in S2 File) of the NCI-60 panel of human cancer cell lines. Predictive models were built for seven drugs often employed in the treatment of ovarian cancer (carboplatin, cisplatin, paclitaxel, docetaxel, gemcitabine, doxorubicin, gefitinib). The drug sensitivities (GI50) of the NCI-60 cell lines approximate a normal distribution (Fig 1A; Fig A in S1 File). For our learning dataset, we conservatively excluded cell lines displaying GI50 values within ±0.50 SD of the mean. The test dataset, however, was selected from all cell lines. In all cases, cell lines used to build the models were distinct from those used in testing the models.

SVM models built upon large datasets typically contain uninformative features, and a number of feature selection methods have been developed to identify subsets of features with optimal predictive accuracy [10–12]. We employed a previously described RFE [13, 14] method to select for features (gene probe sets) that optimally distinguish cells predicted to be sensitive to a drug from those that are not. The RFE method starts by discarding the least relevant features of the model from the bottom of the sorted feature list (Table C in S2 File). The subsequent SVM model is built on the remaining features and again, features with the lowest weights are removed. This process proceeds in a recursive manner until a minimal subset of features is identified that is essential to maintain optimal predictive accuracy. For example, Fig 1B depicts the evolution of predictive accuracy using SVM-RFE feature selection for increased sensitivity to carboplatin (see Fig B in S1 File for feature selection of the other drugs). In this case, initial removal of uninformative features increased accuracy due to the elimination of features that negatively interact with predictive accuracy. Our SVM-RFE approach compares favorably with other commonly employed methods of feature selection (see Fig F in S1 File).

The minimal number of informative features associated with optimally predicted responsiveness to the seven drugs modeled in this study ranged from 10 to 32 (Table D in S2 File). While the biological contribution of the majority of these genes to drug responsiveness is currently unknown, potentially informative trends are often apparent. For example, several of the most informative genes predictive of carboplatin sensitivity have been directly or indirectly implicated with apoptosis (Table E in S2 File), a cellular function known to be induced in response to carboplatin treatment [15].

The SVM models generate drug prediction scores for each cell line. Scores higher than "0" indicate a predicted sensitive response, less than "0" a predicted resistant response (*e*.*g*., see Fig 1C, X-axis). The overall accuracy, specificity and sensitivity are evaluated by leave-one-out cross-validation (LOOCV). The SVM computed predictive scores are plotted against observed GI50 values to graphically display the accuracy of each model. For example, the quadrant plot for carboplatin (Fig 1C) shows that the SVM model is 84% accurate across the NCI-60 test dataset. The predictive accuracies of each of the seven models ranged from 75% to 85% (see Fig C in S1 File for predictive accuracies of the other 6 chemotherapeutic drugs).

### Building SVM-based models across a variety of cancer types improves predictive accuracy

While feature selection methods are designed to identify the most informative features by systematically eliminating less informative ones, the predictive accuracy of ML models is heavily dependent on the presumption that the original learning dataset encompasses the full spectrum of features potentially relevant to the predicted variable [10]. The selection of appropriate learning datasets for building predictive models of cancer drug response is especially challenging because a full understanding of the molecular processes underlying cancer onset/progression has yet to be attained [16]. For this reason, subjective limitations in the scope of data employed in learning datasets may negatively affect predictive accuracy of derived models if informative features are inadvertently excluded. For example, it is frequently assumed that models designed to predict optimal therapies for a particular cancer type should appropriately be built using learning datasets derived exclusively from that same type of cancer. However, a growing body of evidence indicates that the molecular pathways underlying cancer onset/progression are not necessarily defined by a tumor's tissue of origin [17]. Thus, a gene expression pattern associated with a particular cancer type may underlie cancer development in other cancer types as well.

**Fig 2:**
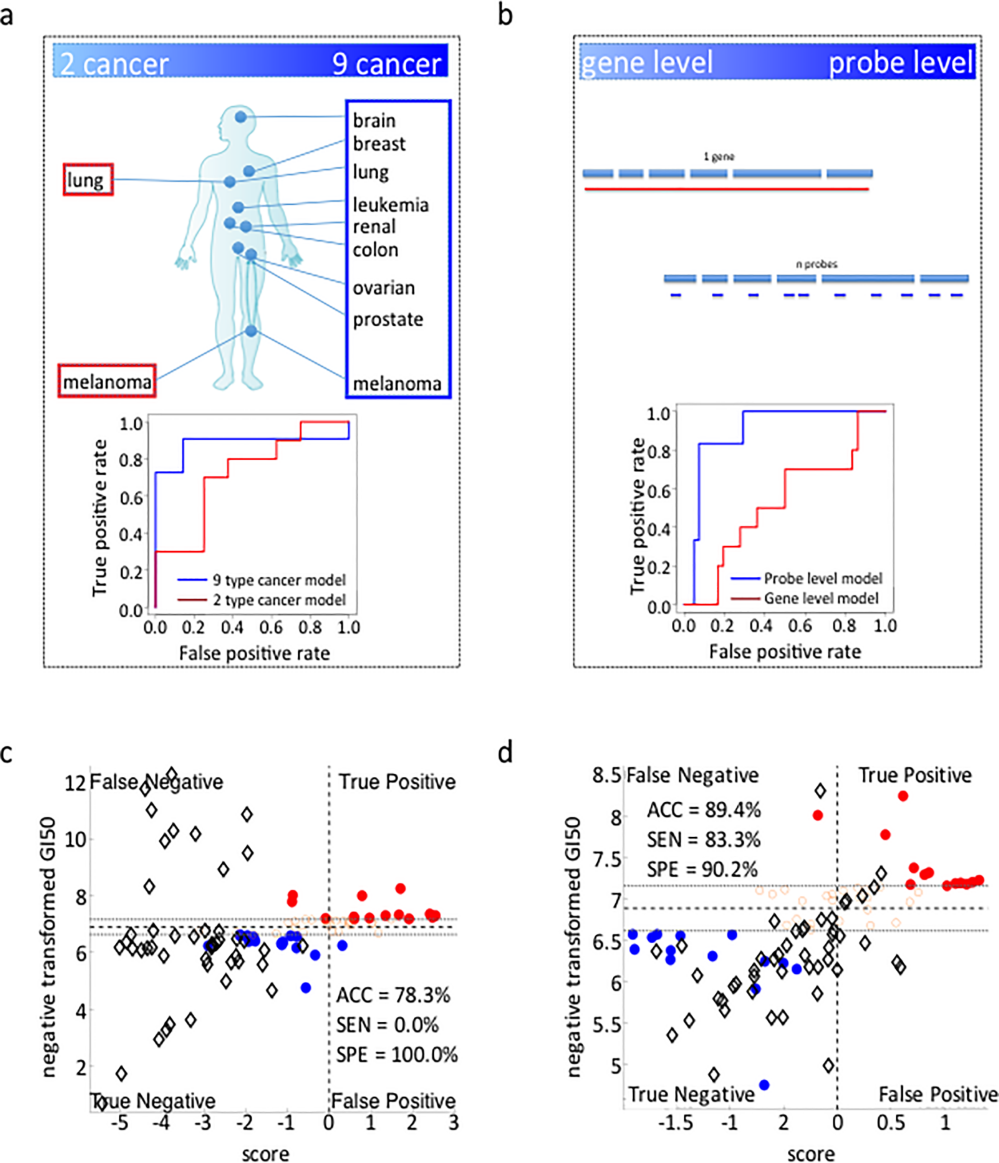
The influence of learning datasets on the predictive accuracy of SVM-RFE models. (A) Comparison of predictive accuracy (ROC curves) for two SVM models of response to carboplatin using a learning dataset derived from 2 cancer types (lung, melanoma) vs. 9 cancer types (brain, breast, lung, leukemia, renal, colon, ovarian, prostate and melanoma). In each case, the data were derived from a total of 18 cell lines. The results indicate that the model built using learning set data from 9 cancer types generates a more accurate prediction (see also Fig D in S1 File); (B,C,D) Prediction of the sensitivity of breast cancer cell lines to doxorubicin. In one case, the model was built using a learning dataset comprised of average gene expression values. In the other case, the model was built using a learning dataset comprised of the expression values of all gene probes. The results demonstrate that the model built using probe set data is more accurate than the model built using average gene expression data; (C) prediction score accuracy using average gene expression values; (D) prediction score accuracy using expression values of all gene probes (Red circles = drug sensitive training set; Blue circles = drug resistant training set; Black diamonds = breast cancer cells test set).

To explore this issue, we compared the relative accuracies of two SVM-derived models designed to predict response to the commonly prescribed cancer drug carboplatin. The respective models were built using gene expression profiles and drug response profiles (*i*.*e*., learning datasets) derived from 18 of the NCI-60 cell lines. In one case, the 18 cell lines were representative of only two types of cancers (lung and melanoma) while in the other case, the 18 cell lines were randomly selected to be representative of all 9 types of cancer comprising the NCI-60 dataset (lung, colon, breast, ovarian, leukemia, renal, melanoma, prostate and CNS). As shown in Fig 2A, the model built using data from the 9 cancer types was more accurate in predicting carboplatin sensitivity (87.5%) than the model built upon only 2 cancer types (75.0%) (Fig D in S1 File). This finding is consistent with growing evidence that the molecular basis of individual cancers may not necessarily be defined by tissue of origin [17]. In addition, the fact that variation in gene expression levels is typically greater among multiple cancer types (see Fig G in S1 File) may be an additional relevant factor since the predictive accuracy of ML models is well known to improve with increasing diversity of the learning set data [10].

### The averaging of microarray probe set expression values reduces predictive accuracy

Another way in which the information content of learning datasets may be compromised is by the employment of average rather than raw experimental values. For example, Affymetrix and other microarray gene expression systems typically incorporate multiple probe sets per gene, thereby providing the possibility of monitoring differences in levels of alternative splicing and other post-transcriptional expression variants (*e*.*g*., Fig E in S1 File). While the use of average gene expression values may be appropriate for many applications, the loss of information associated with the use of such average values in learning datasets could negatively affect the accuracy of drug prediction algorithms if, for example, rare splice variants turn out to be particularly informative features.

To test this possibility, we compared the relative predictive accuracies of two SVM-based algorithms developed to predict the sensitivity of the set of breast cancer cell lines recently employed in the NCI-DREAM Challenge to the drug doxorubicin [1]. In one case, we employed the average Affymetrix gene expression dataset that was provided to the Challenge participants (https://www.synapse.org/#!Synapse:syn2785783). In the other case, we downloaded and employed the original probe data as our learning set (ArrayExpress E-MTAB-181, http://www.ebi.ac.uk/arrayexpress/experiments/E-MTAB-181/). The results presented in Fig 2B, 2C and 2D demonstrate that the model built using the probe set data is substantially more accurate (89%) in predicting the sensitivity of the breast cancer cells lines to doxorubicin than the model (78%) built using the averaged gene expression values.

### Pre-filtering of learning datasets can reduce predictive accuracy

Some methods to assess risk of cancer progression and/or severity focus almost exclusively on genes previously identified as drivers of cancer onset/progression [18]. The advantage of such data pre-filtering is a reduction in the complexity of downstream analyses but, as discussed above, it may also negatively impact the accuracy of derived predictive models if the truncated datasets do not encompass all genes associated with drug sensitivity.

**Fig 3:**
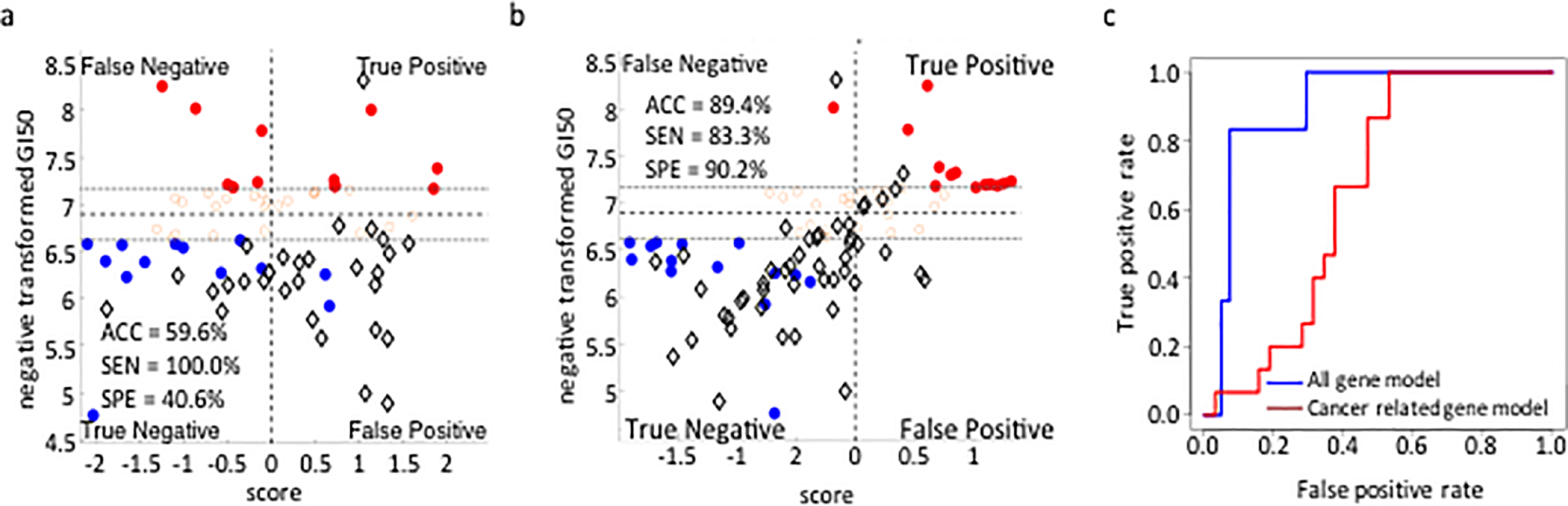
Pre-filtering of learning datasets can reduce the accuracy of predictive models. Shown is the predicted sensitivity of breast cancer cell lines to doxorubicin by two SVM models built using different learning datasets. In one case, the model was built using a learning dataset limited to the expression of 297 genes previously associated with cancer onset/progression [19]. In the other case, the model was built using a learning dataset drawn from all significantly expressed genes (Table A in S2 File). The results indicate that pre-filtering of the learning dataset to only include gene expression values of previously identified cancer related genes reduces predictive accuracy. (A) Quadrant plot of SVM predicted sensitivity to doxorubicin vs. observed sensitivity to doxorubicin of model built using a learning dataset pre-filtered for genes previously associated with cancer onset/progression; (B) Quadrant plot of SVM predicted sensitivity to doxorubicin vs. observed sensitivity to doxorubicin of model built using all gene expression data (Table A in S2 File); (C) ROC curves of the two models showing reduced predictive accuracy associated with the pre-filtered learning dataset (Red circles = drug sensitive training set; Blue circles = drug resistant training set; Black diamonds = breast cancer cells test set).

To explore this question, we compared the predictive accuracy of two SVM-based models using the above breast cancer cell line data. In one model, the learning dataset consisted of expression patterns of 297 genes previously implicated in cancer onset/progression [19] (http://foundationone.com/docs/FoundationOne_tech-info-and-overview.pdf). In the second model, the learning dataset included probes of all significantly expressed genes (Table A in S2 File). The models built using the pre-filtered data from the 297 genes were substantially less accurate (59.6%) in predicting responses to doxorubicin than the model built upon unfiltered data (89.4%) (Fig 3).

### Model applications to human cancer datasets

While our predictive models were established using gene expression and drug sensitivity data from human cell lines, we were interested in conducting preliminary evaluations of the models' ability to predict the response of human cancer patients to chemotherapeutic treatments. Toward this end, we downloaded three independently derived (Affymetrix) gene expression datasets of 273 ovarian cancer patient tumors from the Gene Expression Omnibus (GEO) repository (GSE30161, GSE18521, GSE20565; http://www.ncbi.nlm.nih.gov/gds). The expression values for each individual array were normalized back to the NCI-60 gene expression data matrix.

**Fig 4:**
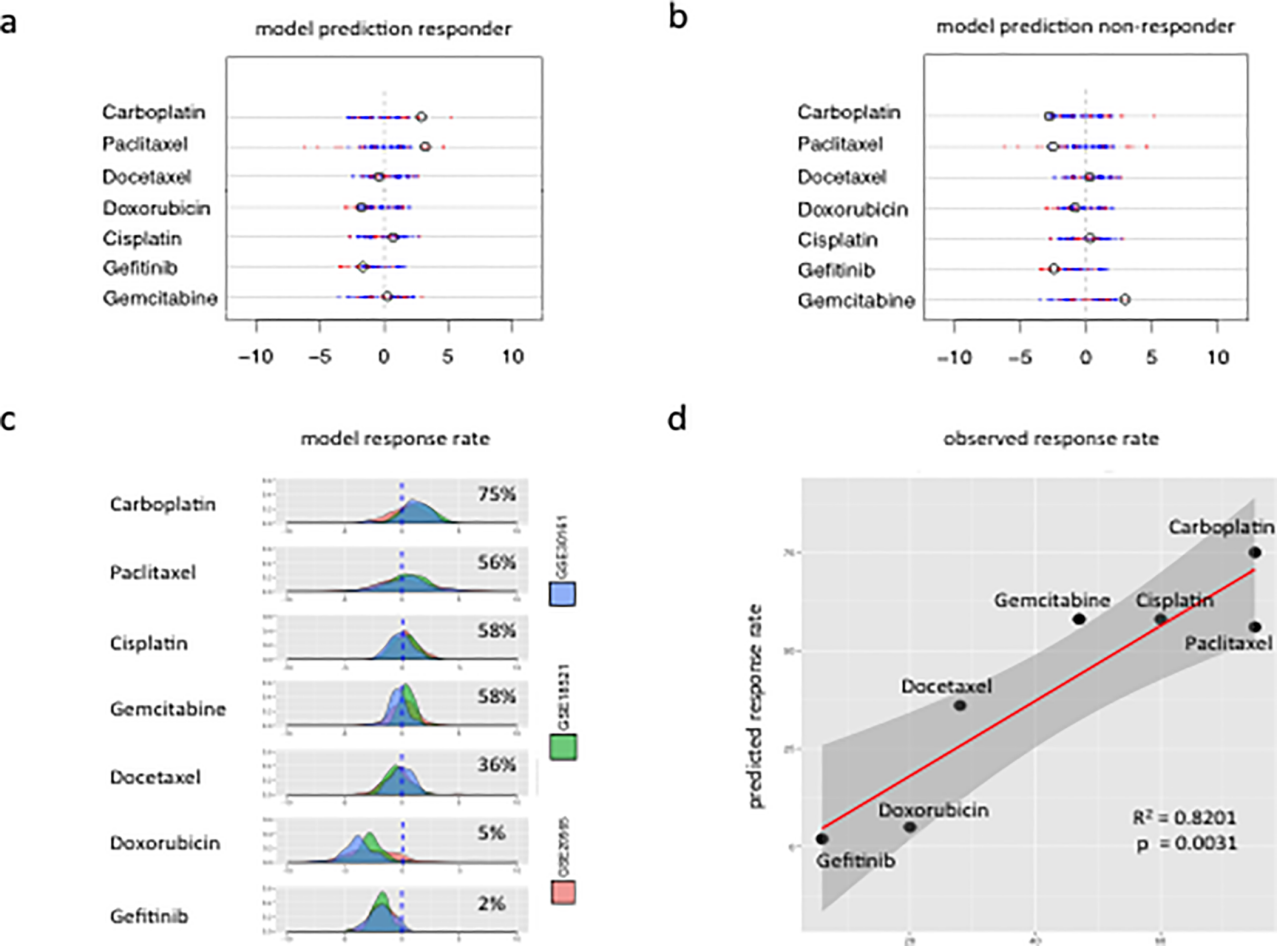
Individual and aggregate prediction of response to chemotherapeutic drugs. The SVM algorithms output binary classifications for each drug (sensitive/resistant) established through a decision function that numerically separates cancer cells predicted to respond to the drug (positive score) from those predicted to be non-responders (negative score). (A) The predicted response of an individual patient (GSM516724) to seven chemotherapeutic drugs. This patient is predicted to respond favorably to the first line therapies of carboplatin (score 2.88) and paclitaxel (score 3.20). (B)The predicted response of a second individual OC patient (GSM516801) to seven chemotherapeutic drugs. The patient is predicted NOT to respond favorably to the first line therapies of carboplatin (score -0.28) and paclitaxel (score -2.53). (C) Density plot of aggregate prediction scores for 3 GEO data sets of 273 ovarian cancer patients and the predicted group response rate for each drug. (D) Scatter plot of the predicted group response rates vs. the observed group responses of OC patients to seven chemotherapeutic drugs (Linear regression p value = 0.0031, R^2^ = 0.8201) (Table F in S2 File).

Using these data, we employed our models to predict the response of the 273 cancer patients to cisplatin, doxorubicin, paclitaxel, carboplatin, docetaxel, gemcitabine and gefitinib. For example, Fig 4A and 4B display the predicted response of two randomly selected patients from the GEO data set. One of the patients (Fig 4A) is predicted to respond favorably to the standard first-line therapy (carboplatin/paclitaxel) while the second patient (Fig 4B) is not. Interestingly, the patient predicted not to respond to first line therapy, is predicted to respond favorably to gemcitabine. Unfortunately, the observed response of these individual patients to therapy is not available. However, the collective response of ovarian cancer patients to the seven drugs analyzed in these studies has been previously reported (Table F in S2 File). To compare the collective predictive accuracy of our models to the collective observed response rates, we combined the predictive sensitivities of the 273 patients comprising the 3 GEO datasets and displayed the results as a distribution of the combined SVM predicted scores (Fig 4C). The results indicate that while at least some patients are predicted to respond to each of the seven drugs, the vast majority (75%) of patients are predicted to respond favorably to carboplatin (Fig 4C), followed closely by gemcitabine, cisplatin (58%) and paclitaxel (56%). Of interest is the fact that carboplatin, given concurrently with paclitaxel, is the current first-line chemotherapy for ovarian cancer patients, with approximately 75–80% of patients being responsive to this combination [20]. Our predictions suggest that the drug primarily responsible for this favorable response is carboplatin. Gemcitabine is commonly given as a second-line chemotherapy for OC and has been found to be of moderate clinical effectiveness, in line with our predictive models [21]. Fig 4D displays the linear regression between the predicted response rates to the seven chemotherapeutic drugs by our models and the observed response rate from clinical studies (Table F in S2 File). The overall predictive accuracy of our models in this dataset is > 80% (R2 = 0.8201).

## Discussion

A primary goal of personalized cancer medicine is the accurate prediction of optimal drug therapies based upon individualized molecular profiles of patient tumors [22]. In an ideal world, such predictions are based upon firmly established cause and effect relationships between identified molecular aberrations and specific aspects of the onset and progression of the disease. An example is the well-established relationship between constitutively active Bcr-Abl tyrosine kinase (TK) expression and the leukemic phenotype associated with CML (chronic myelogenous leukemia) [23]. Patients identified with this molecular aberration are effectively treated with targeted TK inhibitors that work to reduce the elevated activity and restore regulatory balance to the cell. Regrettably, the underlying molecular causes of most tumors are, as yet, not as well understood as for CML. This has led to growing interest in the application of ML approaches to the prediction of optimal drug therapies [18]. ML-based predictive models are not predicated upon knowledge of underlying cause and effect relationships but rather on the identification of significant correlations between specific components of tumor molecular profiles and the favorable response of tumors to specific drugs.

The open source availability of ML prediction algorithms provides the research community with unique opportunities for creative modifications and improvements of existing algorithms not otherwise possible. For example, open sharing of code has been critical to improvements in ML approaches to image recognition [6,7].

Despite the documented advantages of the open sharing of code, to date, the practice has been extremely limited within the field of cancer drug prediction. For example, there is a notable lack of GitHub, Sourceforge, R Bioconductor and other online repositories of cancer drug prediction applications in contrast to the resources available for other ML applications such as the Large Online Image (LOI) repository competitions where alternative computational solutions are openly deposited [7]. We believe that making cancer drug prediction algorithms open source could result in similar benefits in the field of personalized cancer medicine.

Toward that end, we present here an open access support vector machine (SVM)-based algorithm for the predictive response of cancers to seven widely employed chemotherapeutic drugs. The algorithm combines a standard SVM approach with a "one-by one" data normalization pipeline. We have employed the algorithm to explore the effect of a variety of alternative learning datasets on predictive accuracy leading to several unanticipated findings. For example, although it may seem intuitive that drug predictive models for a specific type of cancer should optimally be built upon data from the same cancer type, our results suggest that this may not always be the case. The predictive accuracy of the drug response of a particular cancer type was significantly increased when the model was built using data from a variety of cancer types. This finding is consistent with growing evidence that molecular signatures of optimal cancer drug response are not necessarily defined by the cancer's tissue of origin [17].

Microarray platforms typically monitor gene expression levels using multiple probe sets. This allows discrimination between the expression patterns of alternative splice variants and/or other gene transcript isoforms. Most often, the input expression data for the building of ML predictive models utilizes average expression values across all gene probes. We found that higher accuracy is attained when all probes are incorporated in the learning dataset presumably because some isoforms are more informative than others with respect to drug response and this information is lost or diluted when individual probe data are combined in an average value.

Personal cancer drug therapy, as currently envisioned, involves the targeted inhibition of one or more "cancer driver" genes, *i*.*e*., genes that have been previously identified as playing key roles in cancer onset and progression. For this reason, the molecular profiles of putative cancer driver genes or other pre-defined subsets of genes are often considered sufficient for the accurate prediction of optimal drug therapies. We found that predictive accuracy can, in fact, be significantly reduced when expression profile datasets are pre-filtered prior to ML-based model building. This result suggests that genes involved in cancer drug response are not necessarily limited to those involved in cancer onset even when the drug in question is designed to target a specific group of driver genes.

Although our models were built using the publically available NCI-60 cancer cell line datasets, we are encouraged that predictions using publically available human patient datasets are generally consistent with clinical observations. By making our predictive models open source, we hope to encourage the testing of predictions in additional human datasets representative of a diversity of tumor types.

In summary, our findings demonstrate that significant improvements can be made in the predictive accuracy of ML-based algorithms by modulating the format and/or type of learning datasets employed in the model building process. This finding is likely to be relevant regardless of the type of ML approach employed. While our results illustrate several paths by which the predictive accuracy of our ML-based cancer drug prediction algorithm was improved, these and additional possibilities need to be tested with larger and more extensive datasets. We believe that such goals are most effectively attained by communal efforts where the research community is provided open access to the underlying code and pipelines employed so that meaningful improvements and comparisons with alternative methods can be made [24]. Toward this end, we currently provide an open source R package and pipeline for application of our prediction methods (https://github.com/chuang95/KEA_DrugResponse). In addition, a user-friendly web server is currently under construction that will further enhance public access to our methods.

It is our hope that through community sharing of this and other open source cancer drug prediction algorithms and associated data formatting/normalization procedures that the attainment of a major goal of personalized cancer medicine will be facilitated.

## Materials and methods

### Microarray data normalization

Standard gene expression data analyses were conducted as previously described [25]. Individual gene expression microarray (.CEL) files were normalized one-by-one against the original NCI-60 gene expression microarray data specific to each array (both Affymetrix U133 Plus 2 and Human Exon Array) using standard quantile normalization [26, 27] and using the mean of each probe. This approach creates distributions for each array that are as similar as possible in terms of statistical properties.

### Bifurcation of response data

We define the negative log transformed GI50 value for each cell line. In this approach, the higher the transformed GI50 value, the more sensitive the cell line is to the drug. Three labels were collected: sensitive (marked 1), resistant (marked -1) and indeterminate (marked 0). The sensitive label indicates GI50 values above *mean+0*.*50 SD* while resistant label indicates GI50 values below *mean+0*.*50 SD*. GI50 values that lie within ±0.50 SD of the mean are marked as indeterminate.

### Machine-Learning. SVM: Recursive feature elimination (RFE)

The microarray gene expression values of the NCI-60 cell lines are formatted as a matrix, and sub-divided into training (75%) and validation (25%) datasets. Each probe of a gene is analyzed as a separate feature for each sample. We applied SVM on training data to get weights for each feature, and sort the features based on the weights (Table C in S2 File). Models are built using a learning dataset, and evaluated using a test dataset. Linear support vector machine (SVM) is employed recursively as a classification model to separate samples into two classes: sensitive and resistant. The learning function is *svmtrain* (Matlab R2013b version 8.2.0.701), and the kernel function is linear. The samples are represented as a vector *x*, and the two classes are divided in the dataspace by a hyperplane *wx’ + b = 0* that maximizes the margins between the learning samples of the two classes. This margin is defined such that:

$$\begin{array}{l} wx'+b\geq 1,c=1\\ wx'+b\leq -1,c=-1 \end{array}$$

Binary classification is performed for the test prediction. The prediction score for test samples are calculated by using the decision function as follows:

$$prediction\_socre=-1\times (\sum_{f=1}^{i}w_{f}x_{f}+b)$$

where *w* and *b* are the weight vector and bias parameters from the SVM model. The input *x* is the normalized test sample gene expression data with RFE selected *i* number of features. The classification of the patient drug response is based on this score. We call a sample a responder to the drug if this score is higher than 0, and a non-responder to the drug if the score is lower than 0.

**Fig 5:**
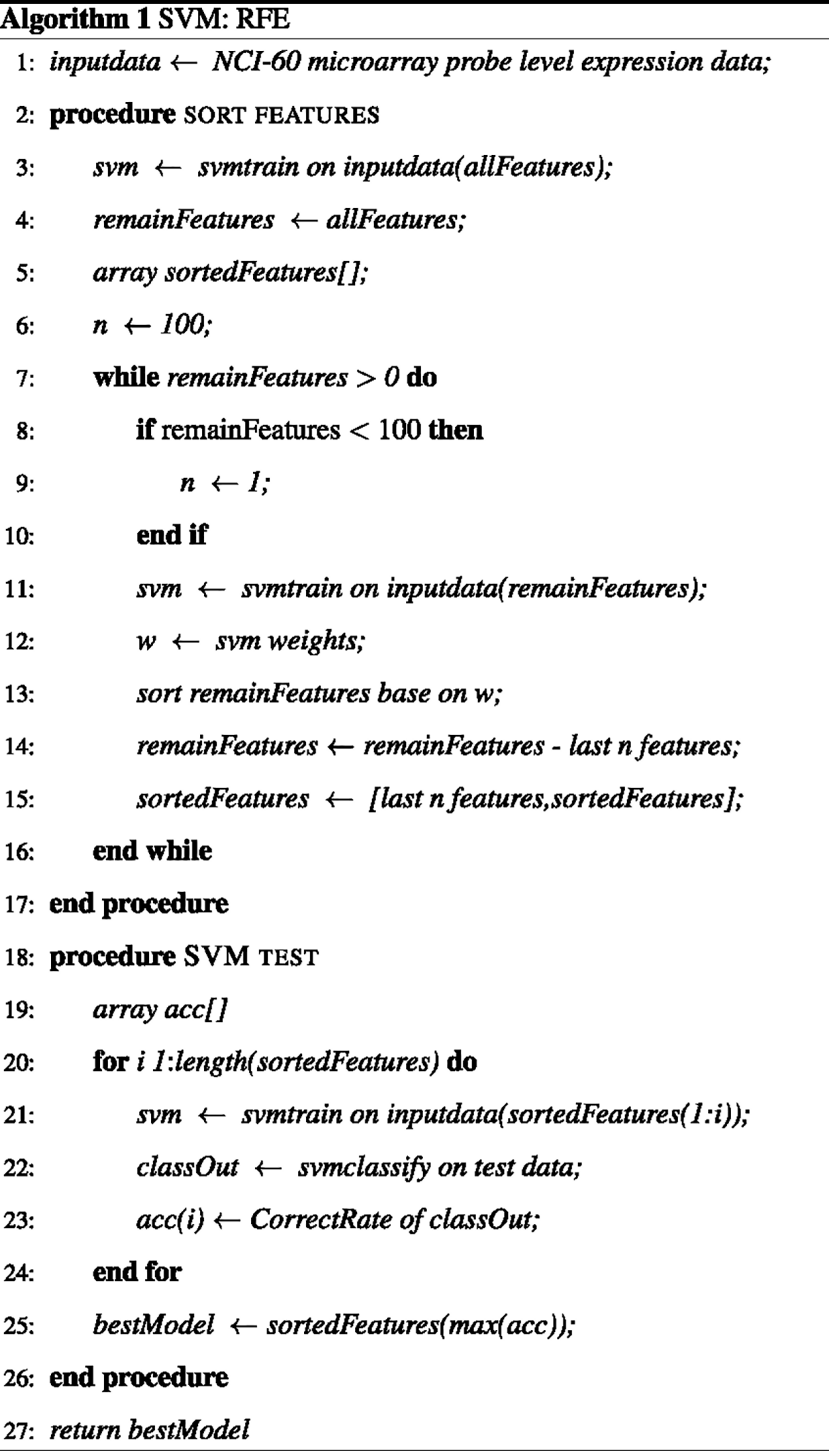
Pseudo code for the RFE approach. This approach takes the microarray expression data of NCI-60 cancer cell lines as input data, and the output is a model with the most informative features.

Recursive feature elimination (RFE) was performed to find the minimum set of features that maximized accuracy in the classification on the test dataset (Fig 5). The approach starts by removing the least relevant 100 features for the model from the bottom (lowest weights) of the sorted feature list. The following SVM model is built using the remaining features, and then again removes the 100 features with lowest weights. This process proceeds recursively until the number of remaining features reaches 100. Thereafter, features are removed one at a time until the most informative set of features is obtained [28–30]. If there are multiple models with the highest accuracy, the model with the fewest number of features is selected. Each model is forced to contain a minimum of ten probes. The predictive model for each drug is based on the most informative set of features determined for that drug. Leave one out cross-validation (LOOCV) is used to evaluate the performance of each of the models as previously described [14].

### Receiver operating characteristic (ROC)

ROC curves were generated using the standard function as outlined below:

AUC=(tpr−fpr+1)/2=(tpr+tnr)/2=1−(fpr+fnr)/2

where AUC = *area under the curve*, tpr = *true positive*, fpr = *false positive*, tnr = *true negative* and fnr = *false negative*. We optimize AUC by maximizing *tpr—fpr* or minimizing a sum of (absolute) normalized error *fpr + fnr*. Optimal models are associated with higher *AUC* values.

## Supporting information

S1 File Supporting information with additional figures. **Fig A**. Ranked display of -log transformed GI50 values for the other six chemotherapeutic drugs for each of the NCI-60 cell lines.
**Fig B**. Evolution of accuracy of predicted drug responses for the other six chemotherapeutic drugs using SVM-RFE selection for gene probe classifiers.
**Fig C**. Visualization of the optimal separation between drug sensitive and resistant NCI-60 cell lines for the other six chemotherapeutic drugs.
**Fig D**. The models built using data from the 9 cancer types vs. 2 cancer types.
**Fig E**. Example of expression levels for probes from the same gene, *NEAT1*.
**Fig F.** Comparison of LOO-cross validation of predicted response to carboplatin using our SVM-RFE method vs. two other commonly employed methods.
**Fig G.** Comparison of the average gene expression for the learning datasets derived from 2 cancer types (lung, melanoma) vs. 9 cancer types (brain, breast, lung, leukemia, renal, colon, ovarian, prostate and melanoma).
(PDF)

S2 File Supporting information with additional tables. **Table A.** Expression values of gene probes (Affymetrix_U133_2.0_plus) for the NCI-60 cell lines.
**Table B.** Sensitivity (GI50) of NCI-60 cell lines to seven chemotherapeutic drugs.
**Table C.** Ranking of probe (feature) weights for employed in the recursive feature elimination (RFE) process for the prediction of carboplatin sensitivity.
**Table D.** Probes associated with optimal predictive accuracy for 7 chemotherapeutic drugs.
**Table E.** Several of the most informative genes predictive of carboplatin sensitivity have been directly or indirectly implicated with apoptosis.
**Table F.** Comparison between predicted response rates of ovarian cancer patients to 7 chemotherapeutic drugs and response rates as reported in the literature.
(XLSX)
