# Supplementary material for: Open source machine-learning algorithms for the prediction of optimal cancer drug therapies
Source: PLoS One. 2017 Oct 26;12(10):e0186906. doi: 10.1371/journal.pone.0186906 (PMC5658085; doi:10.1371/journal.pone.0186906)
Supplement: S1 File — Fig A. Ranked display of -log transformed GI50 values for the other six chemotherapeutic drugs for each of the NCI-60 cell lines. Fig B. Evolution of accuracy of predicted drug responses for the other six chemotherapeutic drugs using SVM-RFE selection for gene probe classifiers. Fig C. Visualization of the optimal separation between drug sensitive and resistant NCI-60 cell lines for the other six chemotherapeutic drugs. Fig D. The models built using data from the 9 cancer types vs. 2 cancer types. Fig E. Example of expression levels for probes from the same gene, NEAT1. Fig F. Comparison of LOO-cross validation of predicted response to carboplatin using our SVM-RFE method vs. two other commonly employed methods. Fig G. Comparison of the average gene expression for the learning datasets derived from 2 cancer types (lung, melanoma) vs. 9 cancer types (brain, breast, lung, leukemia, renal, colon, ovarian, prostate and melanoma). (PDF) [file pone.0186906.s001.pdf]

S1 File for: Open source machine learning algorithms for the prediction of optimal cancer drug therapies

Cai Huang[1,2], Roman Mezencev[1,2], John F. McDonald[¶ 1,2], Fredrik Vannberg[¶ *,1,2]

[1] *School of Biological Sciences, Georgia Institute of Technology, Atlanta GA 30332, USA*
[2] *Parker H. Petit Institute for Bioengineering and Bioscience, Georgia Institute of Technology, Atlanta GA 30332, USA*

[¶] These authors contributed equally to the study

*Correspondence: vannberg@gatech.edu

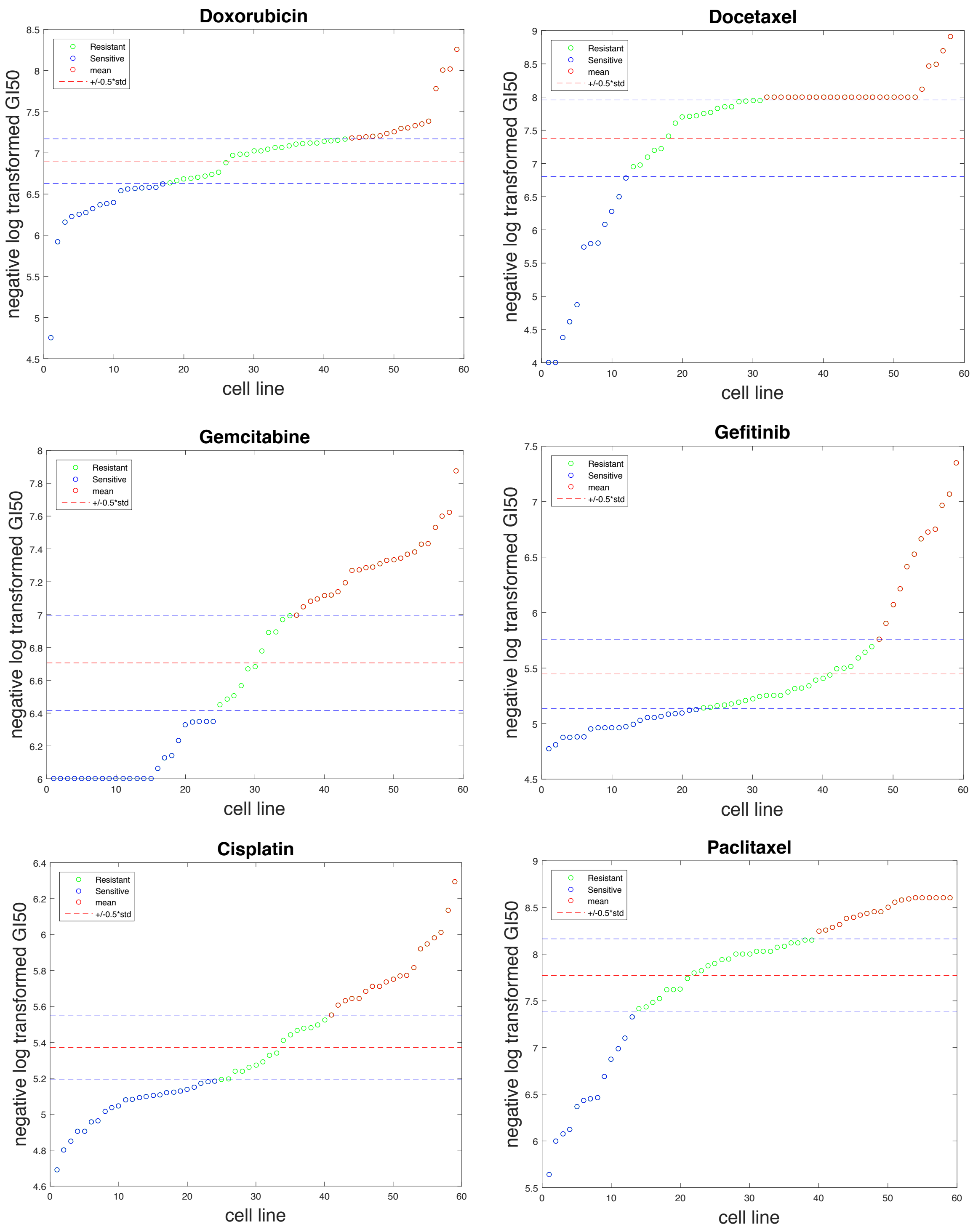

Figure A. Ranked display of -log transformed GI50 values for the other six chemotherapeutic drugs for each of the NCI-60 cell lines. Blue circles = carboplatin resistant cells; red circles = carboplatin sensitive cell lines. Cell lines with GI50 values within ±0.5 SD of the mean (green circles) are less reliably classified as resistant or sensitive and were not employed in learning datasets. Test sets were selected from cells across the entire distribution.

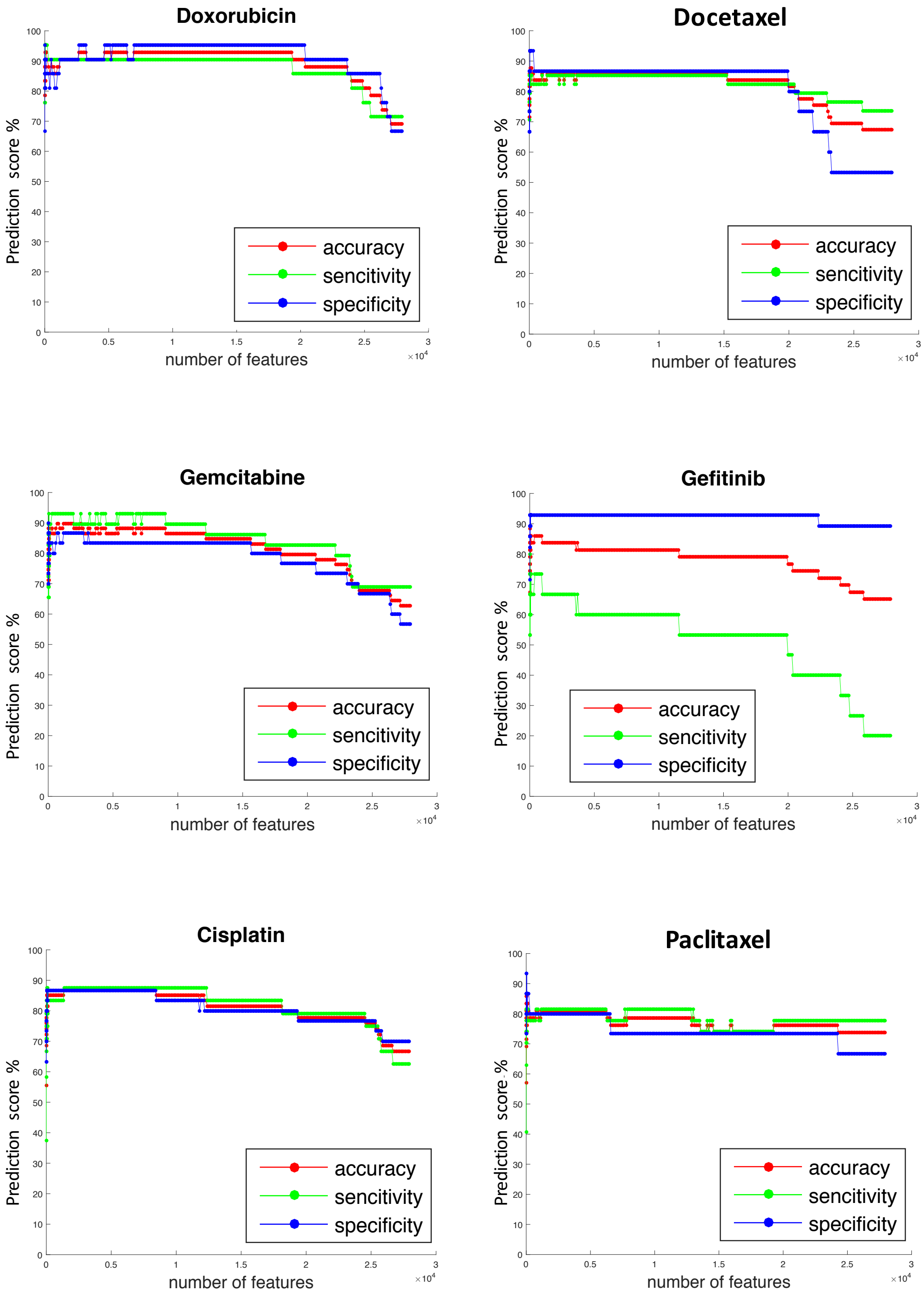

Figure B: Evolution of accuracy of predicted drug responses for the other six chemotherapeutic drugs using SVM-RFE selection for gene probe classifiers.

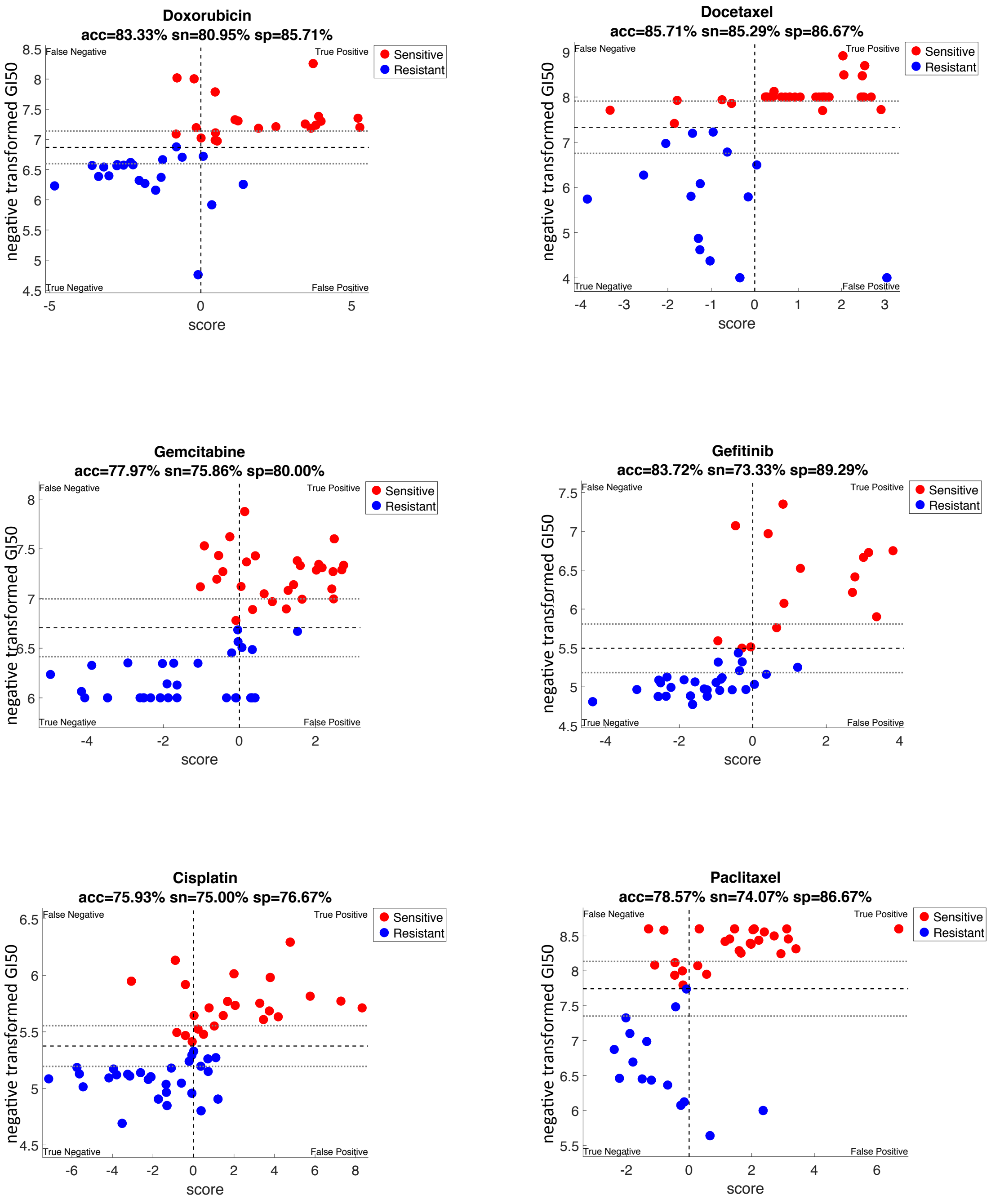

Figure C: Visualization of the optimal separation between drug sensitive and resistant NCI-60 cell lines for the other six chemotherapeutic drugs. The X-axis is the optimal weight vector (prediction score) of the SVM model for carboplatin; the Y-axis is the -log transformed GI50 values for carboplatin.

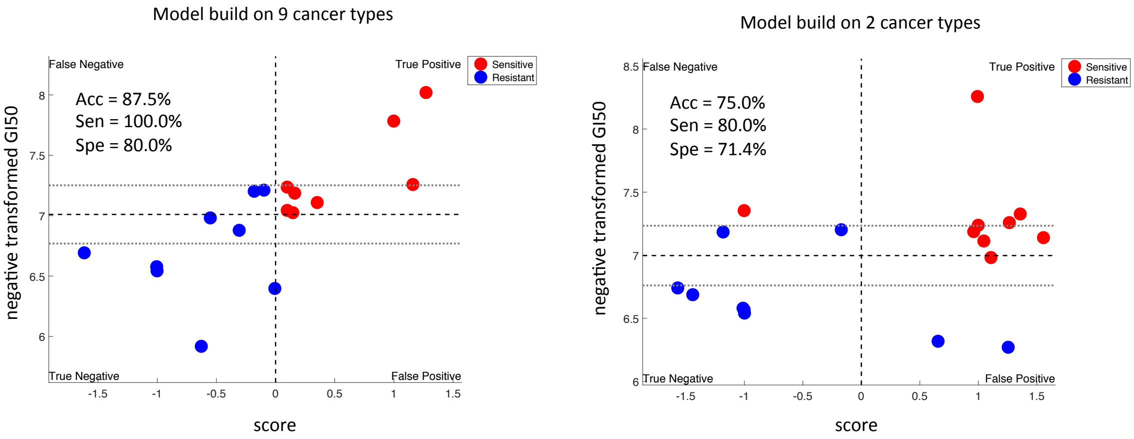

Figure D: The model built using data from the 9 cancer types is more accurate in predicting carboplatin sensitivity (87.5%) than the model built upon only 2 cancer types (75.0%).

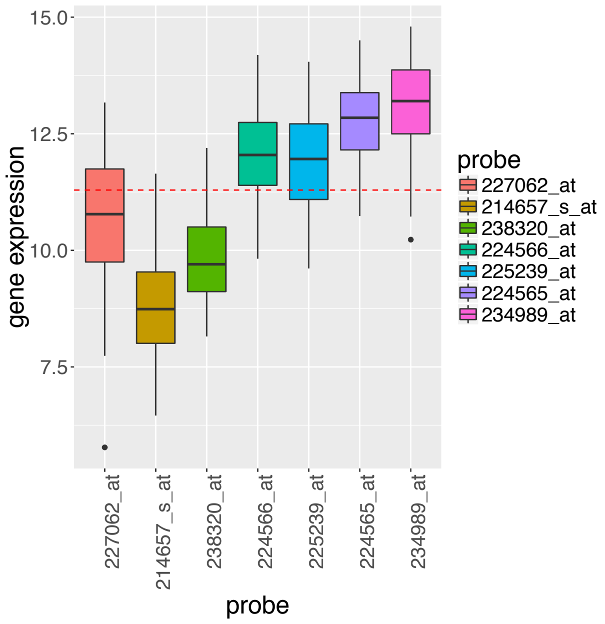

Figure E: The Affymetrix microarray gene expression systems typically incorporate multiple probe sets per gene, thereby providing the possibility of monitoring differences in levels of alternative splicing and other post-transcriptional expression variants. For example, consider the gene *NEAT1* (Nuclear Paraspeckle Assembly Transcript 1) that is represented on the Affymetrix U133 chip by 7 probes. The gene expression associated with this gene varies considerably among probe sets. Relevant information can be lost when a single average value (red dashed line) is presented per gene. In our SVM analysis all probe sets are included in the feature set.

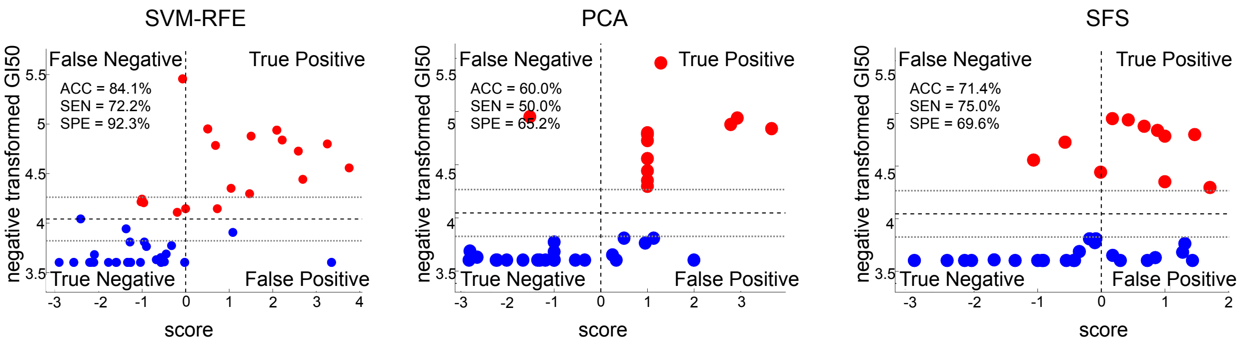

Figure F: Comparison of LOO-cross validation of predicted response to carboplatin using our SVM-RFE method vs. two other commonly employed methods (PCA-Principle Component Analysis; SFS-Sequential Forward Selection). The results demonstrate that the accuracy of the SVM is comparable or superior to other methods.

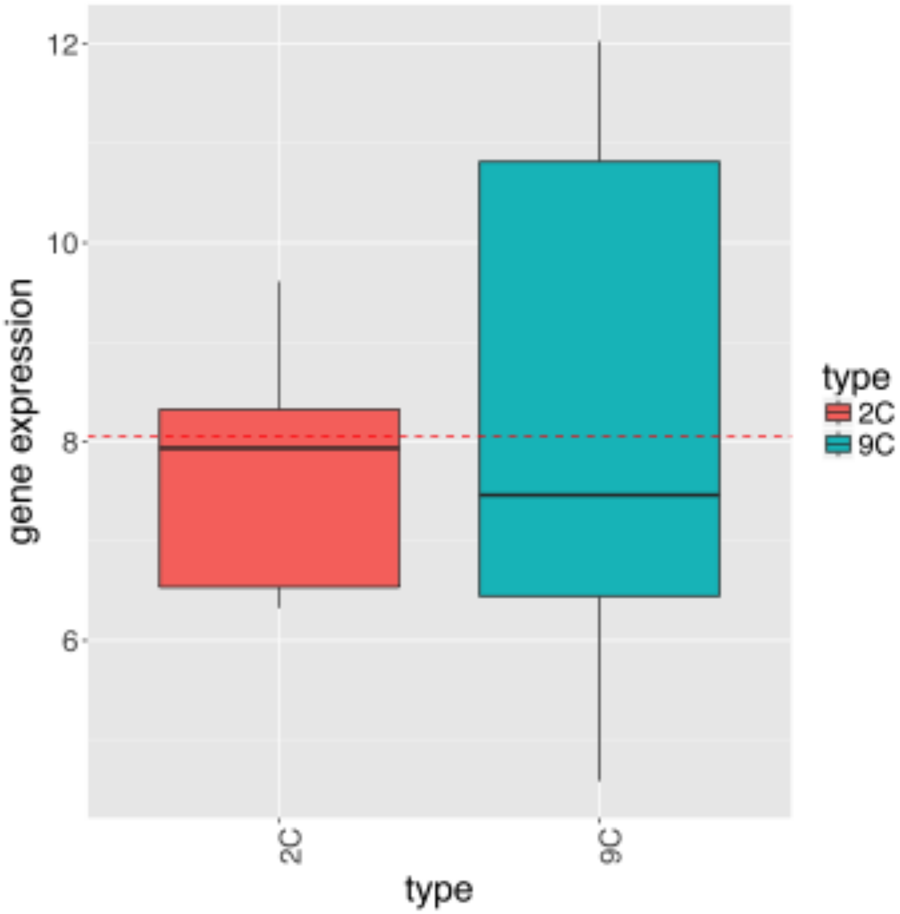

Figure G: Comparison of the average gene expression for the learning datasets derived from 2 cancer types (lung, melanoma) vs. 9 cancer types (brain, breast, lung, leukemia, renal, colon, ovarian, prostate and melanoma). In each case, the data were derived from a total of 18 cell lines, and gene expression values of selected probes are averaged among these 18 cell lines. The results demonstrate that variation in gene expression levels among the 9 cancer types (9C) is significantly greater than between the 2 cancer types (2C).
